# Beyond ACEs and PCEs: Relational Epidemiology and the Childhood Wellness Framework

**DOI:** 10.3390/children13091172

**Published:** 2026-08-31

**Authors:** Jennifer Leason

**Affiliations:** 1 Department of Political Science, Faculty of Arts, University of Calgary, Calgary, AB T2N 1N4, Canada; jennifer.leason@ucalgary.ca; Tel.: +1-587-839-6488; 2 Department of Epidemiology and Biostatistics, University of Western Ontario, London, ON N6G 2M1, Canada; 3 Minegoziibe Anishinaabe Nation, Camperville, MN R0L 0J0, Canada

**Keywords:** Relational Epidemiology, accountability science, childhood wellness, child health, epidemiology, Indigenous health, First Nations, Métis, Inuit, adverse childhood experiences, positive childhood experiences, Indigenous methodologies, feminist ethics of care, child development, health equity, Indigenous rights, self-determination

## Abstract

**Highlights:**

**What are the main findings?**

- This paper proposes Relational Epidemiology, a theoretical orientation that shifts epidemiological inquiry from measuring children’s experiences to evaluating the relationships, systems, and collective responsibilities that shape childhood.
- The Childhood Wellness Framework operationalizes Relational Epidemiology by organizing childhood around collective responsibilities and the conditions necessary for children to flourish, rather than around individual experiences of adversity or resilience.

**What are the implications of the main findings?**

- Relational Epidemiology extends ACEs and PCEs frameworks by expanding epidemiological measurement beyond childhood experiences to include accountability for the systems, institutions, policies, and environments that create childhood conditions.
- Grounded in Indigenous ways of knowing and feminist ethics of care, the Childhood Wellness Framework provides a conceptual foundation for advancing research, clinical practice, public health, policy, and Indigenous self-determination through accountability-oriented approaches to child health.

**Abstract:**

**Background/Objectives:** Adverse Childhood Experiences (ACEs) and Positive Childhood Experiences (PCEs) have fundamentally advanced child health research by demonstrating that early life experiences shape health across the life course. While these frameworks have transformed understanding of trauma, resilience, and child development, they primarily focus on measuring children’s experiences rather than the relationships, systems, and collective responsibilities that create the conditions in which childhood unfolds. This paper proposes Relational Epidemiology as an accountability-oriented expansion of epidemiology and introduces a Childhood Wellness Framework that shifts the scientific gaze from measuring children’s experiences to evaluating the conditions that enable children to thrive. **Methods:** This conceptual paper integrates epidemiological theory, Indigenous ways of knowing, feminist ethics of care, child development, decolonizing methodologies, and Indigenous rights frameworks to examine the assumptions underlying child-centered approaches to measurement and to develop a relational model of childhood wellness. **Results:** Relational Epidemiology is presented as a theoretical orientation that expands epidemiological inquiry beyond describing adversity and resilience toward evaluating the relationships, systems, institutions, and policies that shape childhood. The Childhood Wellness Framework operationalizes this approach by organizing childhood around collective responsibilities and the relational conditions necessary for children to flourish. Grounded in Indigenous knowledge systems and illustrated through the experiences of First Nations, Métis, and Inuit children, the framework demonstrates how accountability can become a measurable focus of child health research. **Conclusions:** Relational Epidemiology extends, rather than replaces, ACEs and PCEs frameworks by shifting the unit of analysis from children’s experiences to the fulfillment of collective responsibilities. The Childhood Wellness Framework offers a conceptual foundation for advancing research, clinical practice, public health, and policy by evaluating not only child outcomes, but also whether societies are creating the conditions necessary for all children to flourish.

## 1. Introduction

*“We have a history of people putting Māori [Indigenous peoples] under a microscope in the same way a scientist looks at an insect. The ones doing the looking are giving themselves the power to define”*

(Merata Mita, as cited in Smith, 2021, [1], p. 67)

For more than two decades, this observation by Māori filmmaker and scholar Merata Mita has challenged researchers to reflect not only on what is studied, but also on who holds the power to define the questions, interpret the findings, and shape the narratives that emerge from research. Although Mita’s critique was directed toward Indigenous research more broadly, it raises an equally important question for contemporary child health research: who or what is placed under the scientific microscope?

Advances in epidemiology, developmental science, and life-course research have demonstrated that childhood experiences profoundly influence physical health, mental health, educational attainment, and wellbeing throughout adulthood [2,3]. This work has fundamentally transformed child health by recognizing childhood as a critical period for intervention and prevention, leading to major advances in maternal and child health, early childhood development, injury prevention, mental health, and public health policy [4,5].

One of the most influential developments in this evolution was the introduction of the Adverse Childhood Experiences (ACEs) Study by Felitti and colleagues [6]. The ACEs framework fundamentally changed how researchers, clinicians, and policymakers understand the long-term consequences of childhood adversity by demonstrating that experiences such as abuse, neglect, and household dysfunction are associated with increased risks of chronic disease, mental illness, substance use, and premature mortality across the life course [6,7]. More recently, Positive Childhood Experiences (PCEs) expanded this perspective by demonstrating that supportive relationships, emotional safety, belonging, and community connectedness can buffer adversity and promote resilience [8,9]. Together, ACEs and PCEs have transformed child health research, informed trauma-informed care, and strengthened understanding of both adversity and resilience. The purpose of this paper is not to question these contributions. Rather, it asks a different question.

Despite asking different questions, ACEs and PCEs share an important methodological assumption: the child remains the primary object of inquiry. ACEs ask, what happened to this child? PCEs ask, what positive experiences did this child have? Both questions have generated important knowledge. Both have improved child health research and practice. Yet both continue to direct the scientific gaze toward children’s experiences rather than toward the broader systems, institutions, relationships, and policies that create the conditions in which those experiences occur.

This distinction is particularly important in Indigenous child health. For First Nations, Métis, and Inuit children, childhood cannot be separated from colonialism, Indigenous rights, self-determination, family, kinship, language, land, culture, and community. Indigenous children do not simply experience adversity or resilience as individual phenomena; they experience the consequences of historical and contemporary decisions made by governments, health systems, education systems, child welfare agencies, and other institutions that shape the conditions of everyday life. Consequently, documenting children’s experiences alone may be insufficient for understanding or changing the conditions that produce them.

What if, instead of placing children under the scientific microscope, we turned the microscope around? What if epidemiology asked not only what happened to this child, but also what responsibilities created the conditions in which this child lives? What if governments, institutions, health systems, researchers, and communities became subjects of systematic inquiry alongside the children whose health they seek to improve?

This paper argues that answering these questions requires a new theoretical orientation within Indigenous epidemiology. We propose Indigenous epidemiology as accountability science, an approach that extends epidemiological inquiry beyond describing health outcomes and childhood experiences to evaluating whether the collective responsibilities of families, communities, institutions, governments, and health systems are being fulfilled. To illustrate this shift, this paper introduces the Childhood Wellness Framework, not as another screening instrument or cumulative risk score, but as a conceptual framework that reorients child health research from measuring children toward measuring the conditions, relationships, and responsibilities that shape childhood. In doing so, this paper demonstrates that Indigenous methodologies do not simply offer a more culturally relevant approach to Indigenous child health; they offer a broader contribution to epidemiology by reframing the purpose of measurement itself, from documenting disparities to advancing accountability for the conditions in which all children are born, grow, belong, and flourish.

## 2. Methods

This study employed a conceptual framework development approach to develop Relational Epidemiology and the Childhood Wellness Framework as an accountability-oriented approach to child health research. Rather than reporting primary empirical findings, this paper synthesizes an interdisciplinary theory to examine the assumptions underlying current approaches to measuring childhood experiences and to propose an alternative conceptual orientation for understanding childhood wellness. Conceptual papers play an important role in advancing scientific knowledge by integrating existing theory, identifying gaps, and developing new theoretical perspectives that can inform future research, policy, and practice [10,11].

### 2.1. Conceptual Approach

This framework was developed through an integrative conceptual synthesis informed by multiple bodies of research, including epidemiology, child development, Adverse Childhood Experiences (ACEs), Positive Childhood Experiences (PCEs), Indigenous methodologies, decolonizing scholarship, feminist ethics of care, population health, and Indigenous rights. This study used an integrative conceptual synthesis to develop Relational Epidemiology and the Childhood Wellness Framework as an accountability-oriented approach to child health research. The purpose was not to conduct a systematic or exhaustive review of the literature, but to bring several bodies of scholarship into conceptual dialog in order to examine how childhood, health, relationality, responsibility, and accountability are understood across different intellectual traditions. Rather than conducting a systematic review, the paper draws upon seminal theoretical and conceptual literature to identify underlying assumptions regarding childhood, health, measurement, and accountability, and to integrate these perspectives into a coherent conceptual framework [10,12].

The conceptual synthesis drew upon scholarship in epidemiology, life-course and child development, Adverse Childhood Experiences (ACEs), Positive Childhood Experiences (PCEs), Indigenous methodologies and relational ways of knowing, decolonizing scholarship, feminist ethics of care, population and structural determinants of health, and Indigenous rights and self-determination. These bodies of scholarship were included because each addresses, from different epistemological and disciplinary locations, questions concerning how health and wellbeing are produced through relationships, environments, institutions, systems, and social conditions.

Framework development proceeded through an iterative process of conceptual comparison and synthesis. First, recurring concepts relevant to childhood wellness and the production of health were identified across these studies. These included relationality, reciprocity, care, belonging, collective responsibility, family and kinship, land and environment, cultural continuity, governance, structural conditions, institutional responsibility, and self-determination. Second, these concepts were compared across bodies of scholarship to identify areas of convergence, distinction, and tension. Particular attention was given to the ways Indigenous relational approaches shift attention from the individual as an isolated unit of analysis toward relationships, responsibilities, and the conditions through which wellbeing is produced.

Third, the recurring concepts were organized around a central accountability question: what relationships, responsibilities, systems, and conditions must be present for children to flourish, and who holds responsibility for creating and sustaining those conditions? This question informed the development of Relational Epidemiology as the broader theoretical orientation of the paper. Concepts were then clustered into domains of collective responsibility that formed the Childhood Wellness Framework, including responsibilities related to welcoming life; love and belonging; family and kinship; language, identity, and culture; land and environment; safe and healthy living conditions; children’s voices; Indigenous self-determination; community and collective wellbeing; and future generations.

The framework was refined iteratively by examining whether each proposed domain shifted the analytic focus away from classifying children’s adversity or resilience and toward evaluating the relationships, systems, institutions, and responsibilities that shape childhood conditions. The resulting framework is therefore not presented as a standardized instrument or exhaustive taxonomy of childhood wellness. Rather, it provides a conceptual structure for asking accountability-oriented questions that may subsequently be examined using epidemiological, qualitative, mixed-methods, policy, Indigenous methodological, or other approaches appropriate to the specific research question and context.

### 2.2. Theoretical Foundations

The conceptual synthesis described above is grounded in two complementary epistemological traditions. First, Indigenous ways of knowing understand children as existing within interconnected relationships [13] extending across family, kinship, community, Nation, land, language [14], culture [15], ancestors, and future generations [16]. Indigenous methodologies [1,17,18] further emphasize relational accountability, reciprocity, and respect as foundational principles for knowledge generation [17,19].

Indigenous methodologies [20] are not simply conventional research methods applied within Indigenous populations or modified for cultural relevance [21]. They arise from Indigenous epistemologies, ontologies, axiologies, and systems of relationship [22] and therefore concern not only how knowledge is collected, but also why knowledge is sought, who has authority to produce and interpret it, to whom researchers are accountable, and how knowledge should benefit communities and Nations. Although Indigenous methodological traditions are diverse and distinctions among First Nations, Métis, Inuit, and other Indigenous Peoples must be respected, recurring principles within Indigenous methodological scholarship include relationality [13,23], relational accountability, reciprocity, respect, responsibility, self-determination, and attention to the relationships through which knowledge is generated and used. In this paper, references to Indigenous relational ways of knowing are not intended to collapse these distinctions. Rather, they identify a recurring orientation within Indigenous scholarship in which persons are understood through relationships extending beyond the autonomous individual to family, kinship, community, Nation, land, language, culture, ancestors, future generations, and the more-than-human world [19]. The meanings, responsibilities, and protocols associated with these relationships remain specific to Peoples, Nations, and contexts. Relational Epidemiology draws particularly upon this understanding of knowledge as occurring within relationships that carry responsibilities.

Indigenous methodologies should also be distinguished from acculturation. Acculturation has commonly been used within social and behavioral research to describe cultural change associated with sustained contact among cultural groups and is frequently operationalized through individual characteristics such as language, identity, affiliation, or cultural practices. Indigenous methodologies instead concern the foundations, relationships, ethics, and governance through which knowledge is produced. Decolonization is similarly distinct from acculturation. In this framework, decolonization refers to challenging colonial structures and assumptions while strengthening Indigenous knowledge systems, jurisdiction, governance, cultural continuity, and self-determination. Neither Indigenous knowledge nor decolonization can therefore be reduced to an individual measure of cultural affiliation.

Second, feminist ethics of care [24] recognizes that human wellbeing develops through relationships of care, interdependence, and mutual responsibility rather than individual autonomy [25,26]. Feminist ethics of care provides an additional intellectual tradition that challenges assumptions of autonomous individualism by emphasizing care, interdependence, vulnerability, and relational responsibility. Important areas of convergence therefore exist between feminist ethics of care and Indigenous relational thought. However, these traditions arise from different intellectual, political, legal, and historical contexts and should not be treated as interchangeable. Indigenous relational obligations, rights, governance, and self-determination are grounded within distinct Indigenous knowledge, legal, cultural, and political traditions and do not derive their legitimacy from feminist ethics of care. In this paper, feminist ethics of care is therefore placed in dialog with Indigenous relational thought rather than positioned as the framework through which Indigenous relationality is interpreted or validated.

There are important areas of convergence between feminist ethics of care and Indigenous relational thought, particularly their rejection of atomistic individualism and their emphasis on interdependence, responsibility, relationships, and the conditions required for flourishing. These similarities, however, should not be interpreted as epistemological equivalence. Indigenous relational knowledge arises from distinct Indigenous intellectual, legal, cultural, territorial, and political traditions and is inseparable from histories and contemporary realities of colonialism, Indigenous rights, governance, and self-determination. Indigenous relationality does not require feminist ethics of care, or another Western theoretical tradition, to establish its legitimacy.

The two traditions are therefore placed in dialog rather than synthesized into a single epistemology. Feminist ethics of care contributes an additional language for examining how care and responsibility become socially and politically distributed, while Indigenous relational approaches provide the foundational orientation through which Relational Epidemiology understands relationships as carrying responsibilities and accountability. Maintaining this distinction is important because conceptual convergence can otherwise obscure differences in histories, power, jurisdiction, and the political and legal status of Indigenous Peoples.

Together, these traditions challenge individualistic assumptions commonly embedded within health research and instead position relationships as the primary context through which children’s wellbeing emerges. These perspectives were integrated with epidemiological theory, including scholarship on population health, social determinants of health [27], life-course epidemiology, and structural determinants of health [28,29,30,31]. This synthesis informed the development of Relational Epidemiology, which extends existing epidemiological approaches by proposing that children’s experiences should be understood as indicators of whether societies are fulfilling their collective responsibilities. Relational Epidemiology also shares points of convergence with Relational Developmental Systems approaches, which conceptualize human development as emerging through dynamic and reciprocal relations among individuals and their biological, social, cultural, historical, and ecological contexts across time. Both orientations challenge simple individualistic or reductionist explanations of development. Relational Epidemiology, however, introduces a different epidemiological emphasis. Rather than primarily examining reciprocal individual-context relations as developmental processes, it asks whether identifiable responsibility-holders including governments, institutions, service systems, communities, and other structures are creating and sustaining the conditions necessary for children to flourish. Its distinctive contribution therefore lies in making relationships, responsibilities, institutional actions, and accountability themselves objects of epidemiological inquiry. The analytic question therefore expands from identifying what children experience to examining how those conditions are produced, who holds responsibility and authority to influence them, and whether those responsibilities are being fulfilled.

### 2.3. Scope of the Framework

The Childhood Wellness Framework is illustrated through the experiences of First Nations, Métis, and Inuit children because Indigenous contexts make visible the relationships among colonialism, governance, systems, and child wellbeing [32]. However, the theoretical orientation of Relational Epidemiology has broader relevance. The framework proposes that childhood wellness is fundamentally relational and that the responsibility for creating healthy childhood conditions extends across families, communities, institutions, governments, and society.

### 2.4. Ethical Considerations

This study is a conceptual analysis based exclusively on published literature and theoretical synthesis. It did not involve human participants, secondary data analysis, biological specimens, or animal research and therefore did not require institutional research ethics approval.

### 2.5. Generative Artificial Intelligence (GenAI)

Generative artificial intelligence (GenAI), specifically Microsoft Copilot in Microsoft Word, was used to support language refinement, editing and organization. GenAI did not generate the theoretical contributions presented in this paper. The concepts of Relational Epidemiology and the Childhood Wellness Framework, the interpretation of the literature, and all arguments are the author’s original contributions. All AI-assisted content was critically reviewed, substantially revised, and verified. The author assumes full responsibility for the integrity, originality and accuracy of this manuscript.

## 3. Results

### 3.1. Childhood as the Unit of Epidemiological Inquiry

Epidemiology has long been concerned with understanding the distribution and determinants of health and disease within populations. Traditionally defined as the study of the frequency, distribution, and determinants of health-related states and events, epidemiology seeks not only to describe patterns of disease but also to identify opportunities for prevention and intervention [33,34]. Over the past century, the discipline has evolved from investigating infectious disease outbreaks to examining the complex interactions among biological, behavioral, social, environmental, and structural determinants of health.

Within child health research, epidemiological inquiry has increasingly focused on childhood as a critical period in the life course. Evidence from developmental epidemiology and life-course research demonstrates that early experiences influence health trajectories extending into adulthood, shaping physical health, mental health, educational attainment, and social wellbeing [3,35]. Consequently, childhood has become a central focus for surveillance, prevention, and early intervention initiatives across medicine, psychology, public health, and social policy.

This emphasis on childhood has produced important advances in child health. Surveillance systems have improved the identification of developmental concerns, adverse exposures, chronic disease risk factors, and social determinants of health. Population-based studies have informed vaccination programs, injury prevention, maternal and child health initiatives, early childhood interventions, and policies addressing poverty, nutrition, and education [31]. Increasingly sophisticated epidemiological methods have strengthened the ability to identify children at elevated risk of poor health outcomes and have supported the development of targeted interventions.

Underlying these advances is a fundamental epidemiological assumption: measuring health allows us to improve it. Surveillance, screening, and risk assessment are not ends in themselves; they are intended to generate evidence that informs prevention, resource allocation, policy development, and clinical care. Epidemiological measurement is therefore not an end in itself; its value also lies in generating knowledge that can inform prevention, policy, resource allocation, and action to improve population health [33].

This orientation has been particularly influential in child health, where identifying risk as early as possible has become a central objective. Increasing attention has been directed toward measuring adverse exposures, protective factors, developmental vulnerabilities, and social determinants during childhood with the expectation that earlier identification will enable earlier intervention. The emergence of Adverse Childhood Experiences (ACEs) and Positive Childhood Experiences (PCEs) reflects this broader epidemiological tradition by recognizing childhood as a critical period during which experiences influence health across the life course [8,9].

Yet an important methodological question remains largely unexplored: what happens after measurement? The success of epidemiology is often judged by its capacity to identify risk factors, quantify disparities, and predict health outcomes. Comparatively less attention has been directed toward evaluating whether the institutions, policies, and systems responsible for creating those conditions are themselves changing. Children may be identified as living in poverty, experiencing food insecurity, lacking access to culturally safe health care, or being exposed to racism and violence. These findings are unquestionably important. However, the act of measuring children’s experiences does not, by itself, alter the structural conditions that produce them.

This observation does not diminish the value of epidemiological measurement. Rather, it raises an important question about its purpose. If measurement identifies inequities without systematically evaluating whether governments, institutions, health systems, and communities fulfill their responsibilities to address them, then epidemiology risks becoming exceptionally effective at documenting disparities while remaining less effective at assessing accountability for the conditions that produce them.

This distinction is particularly relevant within Indigenous child health. Indigenous children have been the subjects of extensive epidemiological surveillance documenting disparities in health, education, housing, child welfare involvement, and social determinants of health. These data have been essential in exposing inequities and informing advocacy. Yet the predominant unit of analysis has remained the child and the outcomes children experience. Far less attention has been devoted to systematically measuring the responsibilities, decisions, governance structures, and institutional actions that create the conditions in which those outcomes occur.

The next evolution of Indigenous epidemiology may therefore require not abandoning measurement, but expanding what is measured. Alongside documenting children’s experiences, epidemiology can ask a complementary question: Who is responsible for creating the conditions in which these experiences occur, and how can those responsibilities themselves become objects of scientific inquiry? This shift does not replace epidemiology’s traditional commitment to surveillance and prevention. Rather, it extends epidemiology toward accountability by recognizing that improving child health requires understanding not only what children experience, but also how systems, relationships, and institutions shape those experiences.

### 3.2. The Evolution of Child-Centered Epidemiology

The development of Adverse Childhood Experiences (ACEs) and Positive Childhood Experiences (PCEs) represents one of the most significant advances in child health research over the past three decades. Together, these frameworks have transformed understanding of how childhood experiences influence health throughout the life course and have fundamentally changed clinical practice, public health, education, psychology, and social policy.

The original ACEs study demonstrated that adverse experiences during childhood—including abuse, neglect, and household dysfunction—are strongly associated with increased risks of chronic disease, mental illness, substance use, and premature mortality later in life [6]. Subsequent research has confirmed these findings across diverse populations and settings, establishing childhood adversity as a major determinant of lifelong health and contributing to the widespread adoption of trauma-informed care and prevention strategies [36].

More recently, Positive Childhood Experiences (PCEs) expanded this body of work by demonstrating that supportive relationships, emotional safety, community belonging, and positive social connections can buffer adversity and promote resilience [37]. Rather than focusing exclusively on risk, PCEs highlighted the importance of strengths, protective relationships, and environments that support healthy child development. Together, ACEs and PCEs have encouraged researchers and practitioners to recognize that both adversity and positive experiences shape health trajectories across the life course.

The contributions of these frameworks are substantial. They have improved recognition of childhood trauma, strengthened early intervention strategies, informed public health policy, and reinforced the importance of investing in children and families. Importantly, they have also shifted attention upstream by demonstrating that experiences occurring early in life have lasting biological, psychological, and social consequences.

Yet despite asking different questions, ACEs and PCEs share a common methodological orientation. Both frameworks position the child’s experiences as the primary object of scientific inquiry. ACEs focus on understanding the adversities children experience, while PCEs focus on identifying the positive experiences that promote resilience. Although one emphasizes risk and the other protection, both locate the principal unit of analysis within the child and the experiences the child has encountered.

This observation is not intended as a criticism of either framework. Rather, it highlights an important opportunity to extend child health research. If epidemiology seeks to understand the determinants of health, should inquiry stop at children’s experiences, or should it also examine the systems, policies, relationships, and institutions responsible for creating the conditions in which those experiences occur?

The distinction is subtle but significant. Childhood experiences do not arise in isolation. They emerge within families, communities, schools, health systems, governments, cultures, economies, and environments. Experiences of adversity or belonging, exclusion or safety, instability or security are shaped by decisions made long before a child encounters them. Housing policy influences overcrowding. Education policy influences language revitalization and cultural continuity. Health policy influences access to prenatal care, birth services, mental health support, and preventive care. Child welfare legislation influences family preservation and kinship care. These systems do not simply respond to childhood experiences; they help create the conditions in which those experiences become possible.

From this perspective, childhood experiences may be understood not only as exposures to be measured but also as outcomes of broader social, political, cultural, environmental, and institutional conditions. This raises an important methodological question for epidemiology: should the primary object of inquiry remain the child, or should epidemiology also systematically examine the responsibilities that shape children’s conditions of life?

This final question does not replace ACEs or PCEs. Rather, it extends their contribution by recognizing that childhood experiences are themselves produced within broader systems of responsibility. Understanding what happened to children remains essential. Equally important, however, is understanding why those experiences occurred, who was responsible for preventing them, and whether the institutions responsible for children’s wellbeing are fulfilling their obligations. It is this shift from measuring children’s experiences to measuring collective responsibilities that forms the foundation of this paper.

### 3.3. Relational Epidemiology: An Accountability Orientation for Child Health

Epidemiology has traditionally been defined as the study of the distribution and determinants of health-related states and events within populations and the application of this knowledge to improve health [33]. Across infectious diseases, chronic diseases, environmental health, and population health, epidemiology has sought to identify risk factors, estimate associations, understand causal pathways, and inform prevention. These contributions have fundamentally shaped public health by improving understanding of who experiences disease, where disease occurs, and which exposures influence health outcomes.

Within child health, this orientation has produced profound advances. Epidemiological research has demonstrated that early-life experiences influence health across the life course, thereby strengthening surveillance, prevention, developmental science, and early intervention [35,38]. Frameworks such as Adverse Childhood Experiences (ACEs) and Positive Childhood Experiences (PCEs) have extended this work by documenting how adversity and protective relationships influence children’s lifelong health trajectories [39]. Together, these approaches have transformed child health science.

Yet epidemiology has largely remained grounded in an individualistic orientation. Even when examining social determinants of health, the primary object of inquiry often remains the individual or population experiencing exposure. Children’s experiences become the outcomes to be measured, classified, compared, and explained. This paper argues that epidemiology can be expanded through a different theoretical orientation: Relational Epidemiology.

Relational Epidemiology does not reject conventional epidemiology. Rather, it extends its purpose by recognizing that health is not produced solely through individual exposures or behaviors but emerges through relationships, responsibilities, systems, and the conditions they create. Instead of asking only what happened to individuals, Relational Epidemiology asks how relationships among families, communities, institutions, governments, and environments create the conditions in which health becomes possible. This orientation draws upon two complementary intellectual traditions.

The first is Indigenous ways of knowing. Across First Nations, Métis, Inuit, and other Indigenous knowledge systems, wellbeing is understood as inherently relational. Individuals exist within interconnected relationships with family, community, land, language, culture, ancestors, future generations, and the more-than-human world [17]. Indigenous scholars have argued that colonialism itself functions as a fundamental determinant of health, shaping housing, education, food systems, governance, language, culture, and access to services across generations [40,41,42]. Relational Epidemiology builds upon these insights by proposing that epidemiology move beyond documenting colonial determinants toward evaluating whether governments, institutions, and systems are fulfilling their responsibilities to transform them. Health is therefore not solely an individual outcome but a reflection of the strength of these relationships and of the reciprocal responsibilities that sustain them.

The second is feminist ethics of care. Feminist scholars have long challenged models of human development that privilege autonomy, independence, and individualism, arguing instead that care, interdependence, vulnerability, and relational responsibility are fundamental conditions of human life [25]. Rather than viewing dependency as a weakness, ethics of care recognizes that every person enters and moves through life within networks of care that make survival and flourishing possible [24].

Although these traditions emerge from different intellectual histories, they converge around a shared premise: human wellbeing is fundamentally relational. Children are never independent actors. They are born into relationships, shaped by relationships, and flourish through relationships. Their health reflects not only their individual experiences but also the quality of the systems, communities, environments, and relationships that surround them.

This relational orientation has particular significance for epidemiology. Conventional epidemiology asks what exposures increase risk and what factors protect health. Relational Epidemiology asks an additional question: who is responsible for creating the conditions in which those exposures and protections occur? This question represents more than a methodological refinement; it reorients the purpose of epidemiological inquiry. Childhood adversity is understood not simply as an exposure to be measured but as evidence that relationships, responsibilities, or systems have failed to provide the conditions necessary for children to thrive. Likewise, positive childhood experiences are understood not merely as protective factors but as the expression of relationships, policies, institutions, and communities fulfilling their responsibilities to children.

From this perspective, epidemiology expands beyond documenting disparities toward evaluating accountability. The purpose of measurement is no longer limited to identifying which children experience adversity or resilience, but also to examining whether governments, institutions, health systems, researchers, and communities are creating the conditions that enable children to flourish.

Relational Epidemiology therefore proposes a shift in the scientific gaze. Rather than placing children at the center of inquiry, it positions relationships, responsibilities, and systems as legitimate objects of epidemiological investigation. The central question is no longer simply “What happened to this child?” but “What relationships, responsibilities, and systems created the conditions in which this child lives?”

This orientation does not replace traditional epidemiology; it broadens it. Risk factors remain important. Childhood experiences remain important. Surveillance remains important. However, Relational Epidemiology argues that epidemiology has an additional responsibility: to evaluate not only the distribution of health outcomes but also the fulfillment of the collective responsibilities that shape those outcomes. In doing so, epidemiology moves beyond describing inequities toward supporting accountability, systems transformation, and the conditions necessary for all children to flourish.

### 3.4. Turning the Scientific Gaze: From Measuring Children to Measuring Responsibilities

Merata Mita’s observation that Indigenous peoples have historically been “put under a microscope” remains one of the most enduring critiques of research. Her concern was not simply that Indigenous peoples were studied, but that research positioned them as the objects of inquiry while leaving the structures of power that shaped their lives largely unquestioned [1]. Research became a means of describing Indigenous peoples rather than interrogating the systems that defined, governed, and constrained their lives.

Although child health research has evolved considerably since Mita first articulated this critique, a similar orientation continues to shape contemporary epidemiology. Advances such as Adverse Childhood Experiences (ACEs), Positive Childhood Experiences (PCEs), developmental neuroscience, and life-course epidemiology have fundamentally improved understanding of how childhood influences health across the lifespan. Yet despite their important differences, these approaches continue to direct the primary scientific gaze toward children themselves. Researchers ask what happened to children. Clinicians assess children’s risk. Public health surveillance measures children’s outcomes. Interventions are designed to improve children’s resilience. Children remain the principal objects of inquiry.

This orientation has generated invaluable knowledge. Without ACEs, childhood trauma would likely remain under-recognized within medicine and public health. Without PCEs, the importance of supportive relationships, belonging, and resilience would receive far less attention. These contributions should not be minimized. They have transformed child health research and improved countless lives. The question, however, is not whether these frameworks are valuable. The question is whether they ask enough. What remains outside the frame when the scientific gaze is directed primarily toward children?

When epidemiological inquiry begins with children, the experiences being measured often appear as characteristics of those children: high ACEs scores, developmental vulnerability, poor educational outcomes, mental health challenges, or resilience. Yet these experiences emerge within environments that children neither choose nor control. Housing insecurity, unsafe drinking water, inaccessible health services, systemic racism, environmental degradation, educational inequities, child welfare policies, and the erosion of language and culture are not created by children. They are produced through decisions, institutions, histories, relationships, and systems.

As demonstrated in Table 1, the Childhood Wellness Framework proposes that systems should be recognized as equally legitimate objects of scientific inquiry. Rather than asking only, “What happened to this child?” Relational Epidemiology asks, “Who created the conditions in which this child lives?” This shift does not abandon child-centered care. Rather, it recognizes that meaningful improvements in child wellbeing depend on understanding the relationships and systems that shape children’s everyday conditions. Children’s experiences remain critically important, but they are no longer viewed as the endpoint of inquiry. They serve as indicators of how effectively families, communities, governments, institutions, and societies fulfill their responsibilities to children.

This shift also transforms the purpose of measurement. Measurement is no longer undertaken solely to classify risk or describe disparities. Instead, it becomes a means of evaluating accountability. High rates of food insecurity raise questions about income policy and food systems. Elevated child welfare involvement raises questions about legislation, prevention, housing, and family support. Language loss raises questions about educational policy, colonial histories, and investments in Indigenous language revitalization. Childhood experiences provide evidence of the broader relational conditions in which children grow.

This reorientation represents a fundamental shift in the unit of analysis. Rather than positioning children as the primary subjects of scientific investigation, Relational Epidemiology places relationships, responsibilities, institutions, and systems under the scientific gaze. Children’s wellbeing remains the ultimate outcome, but responsibility for creating that wellbeing is understood as collective rather than individual. This is the central proposition of this paper. The next evolution of child health research is not simply to develop more sophisticated measures of childhood adversity or resilience. It is to develop equally rigorous ways of measuring whether societies are fulfilling their responsibilities to children. In doing so, epidemiology moves beyond documenting childhood experiences toward evaluating the conditions that make childhood flourishing or childhood adversity possible.

### 3.5. The Childhood Wellness Framework: An Operational Model for Relational Epidemiology

If Relational Epidemiology reorients epidemiological inquiry from measuring children to examining the relationships, responsibilities, and systems that shape childhood, an important practical question follows: how can this theoretical orientation inform research, policy, and practice? This paper proposes the Childhood Wellness Framework (CWF) as an operational model for Relational Epidemiology.

The Childhood Wellness Framework is not intended to replace Adverse Childhood Experiences (ACEs) or Positive Childhood Experiences (PCEs). Nor is it proposed as another screening instrument, psychometric scale, cumulative risk score, or diagnostic checklist. Because the Childhood Wellness Framework is not proposed as a standardized scale or child-level assessment instrument, its development should not be interpreted as requiring psychometric validation of the framework as a whole. Rather, empirical applications would require forms of validation and methodological rigor appropriate to the specific responsibility, indicator, data source, research design, and knowledge system being examined. Survey-derived measures may require assessment of reliability and construct validity, while administrative indicators require attention to completeness, accuracy, coverage, and interpretability. Policy- and systems-level measures require transparent specification of the responsibility-holder, obligation, benchmark, timeframe, and evidence of fulfillment. Qualitative, mixed-methods, Indigenous methodological, community-defined, and Nation-defined approaches require standards of rigor and accountability appropriate to those methodologies and governance contexts. Rather, the Childhood Wellness Framework is a conceptual framework that shifts attention from measuring children’s experiences to understanding the relational conditions that enable children to thrive and the collective responsibilities required to sustain those conditions.

This distinction is fundamental. ACEs ask what adverse experiences children have survived. PCEs ask about the positive experiences children have had. The Childhood Wellness Framework asks a different question: what relationships, responsibilities, and conditions are necessary for children to flourish, and who is responsible for creating and sustaining them? This question reflects the theoretical foundations of Relational Epidemiology. Childhood experiences are understood not as isolated events or characteristics of individual children, but as expressions of broader relational, social, cultural, environmental, economic, and political conditions. Consequently, the purpose of inquiry shifts from documenting children’s experiences alone to evaluating whether families, communities, institutions, governments, and health systems are creating environments in which children can flourish.

The framework is grounded in Indigenous ways of knowing and feminist ethics of care. Indigenous knowledge systems understand children as belonging within interconnected relationships extending across family, kinship, community, Nation, land, language, culture, ancestors, and future generations [17]. Feminist ethics of care similarly recognizes that human beings are fundamentally relational, developing within networks of care, interdependence, reciprocity, and shared responsibility rather than as autonomous individuals [26,43]. Although these traditions arise from different intellectual histories, both reject the assumption that wellbeing can be understood solely at the level of the individual. Together, they provide a philosophical foundation for understanding childhood as a relational process rather than an individual state.

Accordingly, the Childhood Wellness Framework organizes childhood around the conditions that support healthy development rather than around categories of adversity or resilience. These conditions include welcoming children into life with dignity; nurturing love, belonging, and secure relationships; strengthening family and kinship; supporting language, identity, and cultural continuity; protecting relationships with land and community; ensuring safety and basic needs; respecting children’s voices; and upholding the rights of children, families, and Nations. Each condition represents both an essential dimension of childhood wellness and a shared societal responsibility extending across families, communities, institutions, governments, and civil society.

Importantly, these domains are not conceptualized as protective factors possessed by individual children. They are relational conditions that societies have an obligation to create, sustain, and protect. In this way, the framework shifts the unit of analysis away from children themselves and toward the relationships and environments that shape children’s opportunities to grow, belong, and flourish.

The Childhood Wellness Framework operationalizes Relational Epidemiology by providing a structured approach to examining accountability across multiple levels of society. Rather than asking only whether children have experienced adversity or resilience, researchers can also examine whether systems are fulfilling their responsibilities to ensure safe births, secure families, thriving communities, cultural continuity, healthy environments, equitable opportunities, and meaningful participation in community life. Childhood wellness thus becomes an indicator of how effectively societies uphold their obligations to children.

If Relational Epidemiology provides the theoretical orientation for understanding childhood as fundamentally relational, then the Childhood Wellness Framework provides its conceptual expression. Grounded in Indigenous ways of knowing and feminist ethics of care, the framework illustrates how relationships, responsibilities, and systems create the conditions through which childhood wellness emerges. Rather than representing a culturally adapted model of existing child health frameworks, the Childhood Wellness Framework demonstrates how Indigenous knowledge can contribute to a broader rethinking of epidemiology itself.

Although this framework is grounded in Indigenous ways of knowing and developed in response to the realities of First Nations, Métis, and Inuit children, its conceptual orientation has broader relevance. Table 2 introduces the Childhood Wellness Framework as an operational model for Relational Epidemiology and principles that may be applied across societies. Children do not flourish because they are inherently resilient; they flourish because relationships, communities, institutions, and governments create the conditions that allow them to do so. In this way, Indigenous knowledge contributes not only to Indigenous child health but also to a broader rethinking of epidemiology by demonstrating that accountability for childhood wellbeing is a collective societal responsibility.

### 3.6. Why Indigenous Contexts Matter

Although the Childhood Wellness Framework is grounded in principles of Relational Epidemiology that have relevance across child health, it is intentionally illustrated through the experiences of First Nations, Métis, and Inuit children. This is not because Indigenous children require an entirely different science of child development. Rather, Indigenous contexts reveal with particular clarity how children’s wellbeing is shaped by relationships, governance, systems, and the fulfillment—or neglect—of collective responsibilities.

For Indigenous children, childhood unfolds within the enduring contexts of colonialism, Indigenous resurgence, and self-determination. Policies affecting land, language, housing, education, child welfare, health care, and jurisdiction are not peripheral influences on child wellbeing; they are central determinants of the conditions in which children are born, grow, belong, and flourish. Historical and contemporary colonial policies have disrupted families, kinship systems, cultural continuity, and community governance, while Indigenous Nations continue to rebuild these foundations through language revitalization, land-based education, Indigenous-led health services, and the exercise of self-determination [32,44].

These realities make Indigenous childhood an especially important context for advancing Relational Epidemiology. Frameworks centered primarily on individual experiences of adversity or resilience may document important outcomes but are less equipped to examine the upstream responsibilities that shape those outcomes. In contrast, Indigenous ways of knowing foreground relationships, reciprocity, collective responsibility, and accountability, making visible the social, cultural, political, and environmental conditions that sustain child wellbeing. The Childhood Wellness Framework is therefore not presented as an Indigenous adaptation of ACEs or PCEs. It is an Indigenous-informed conceptual framework that demonstrates how Relational Epidemiology can shift the unit of analysis from children’s experiences to the conditions and responsibilities that shape those experiences. While developed in partnership with Indigenous knowledge and grounded in the realities of First Nations, Métis, and Inuit children, the framework’s conceptual insights have broader implications for child health research, policy, and practice. Indigenous contexts illuminate principles of relational accountability that can strengthen epidemiological inquiry for all children while remaining rooted in Indigenous rights, knowledge systems, and aspirations for self-determination.

### 3.7. Measuring Collective Responsibilities for Childhood Wellness

If Relational Epidemiology shifts the purpose of epidemiological inquiry from measuring children’s experiences to evaluating the relationships, systems, and conditions that shape childhood, then measurement itself must also change. Rather than organizing inquiry around categories of adversity or resilience, the Childhood Wellness Framework proposes that epidemiology examine the collective responsibilities required to create the conditions in which children can flourish.

This represents a fundamental shift in the unit of analysis. Responsibility is no longer understood as belonging solely to parents or caregivers. Childhood wellness is created through interconnected responsibilities shared across families, extended kinship networks, communities, Nations, governments, institutions, health systems, education systems, and society more broadly. Children’s wellbeing therefore becomes an indicator of whether these responsibilities are being fulfilled. Each responsibility within the Childhood Wellness Framework represents both an essential condition for healthy child development and a question that epidemiology can investigate. Rather than asking what happened to children, Relational Epidemiology asks whether societies have created the conditions necessary for children to thrive.

#### 3.7.1. Responsibility to Welcome Life

Every child has the right to enter life with dignity, safety, love, and appropriate care. This responsibility begins before birth through maternal wellbeing, culturally safe prenatal care, access to midwifery and maternity services, adequate nutrition, secure housing, and freedom from violence. Within Indigenous contexts, this responsibility also includes supporting Indigenous birthing practices, reducing unnecessary birth evacuation, and ensuring that birth occurs, wherever possible, within family, community, language, and culture. Measuring this responsibility requires examining not only birth outcomes but also whether health systems create conditions that honor birth as a relational and cultural event.

#### 3.7.2. Responsibility to Nurture Love and Belonging

Children flourish when they know they are loved, valued, and connected. Love, belonging, and secure attachment are not simply protective factors; they are responsibilities shared across families, communities, and institutions. Relational Epidemiology therefore examines whether children have opportunities to develop stable, caring relationships and whether policies strengthen rather than disrupt family and community connections.

#### 3.7.3. Responsibility to Strengthen Families and Kinship

Children develop within networks of care that extend beyond parents alone. Grandparents, siblings, aunts, uncles, Elders, and chosen family all contribute to childhood wellness. Policies affecting parental leave, housing, income security, child welfare, and family reunification therefore become relevant indicators of whether societies support or undermine children’s relational worlds.

#### 3.7.4. Responsibility to Sustain Language, Identity, and Culture

Language, cultural identity, and cultural continuity are fundamental conditions of childhood wellness. For First Nations, Métis, and Inuit children, opportunities to learn Indigenous languages, participate in ceremony, engage with Elders, and experience cultural teachings contribute to belonging, identity, and wellbeing. Measuring this responsibility requires examining investments in language revitalization, culturally relevant education, community leadership, and opportunities for cultural participation.

#### 3.7.5. Responsibility to Protect Relationships with Land and Environment

Children’s relationships with land influence physical, emotional, spiritual, cultural, and environmental wellbeing. Safe housing, clean water, healthy ecosystems, access to traditional foods, and opportunities to spend time on the land represent important conditions for childhood wellness. In Indigenous contexts, relationships with land are inseparable from identity, governance, and cultural continuity, making environmental stewardship a responsibility for present and future generations.

#### 3.7.6. Responsibility to Ensure Safety and the Conditions for Daily Living

Childhood wellness requires more than protection from harm. It requires access to safe housing, nutritious food, education, health care, recreation, transportation, and economic security. Rather than conceptualizing these as social determinants external to child development, the Childhood Wellness Framework recognizes them as fundamental responsibilities that enable children to live healthy and fulfilling lives.

#### 3.7.7. Responsibility to Honor Children’s Voices

Children are active participants in their own lives and have the right to express their views in ways appropriate to their age, development, and culture. Respecting children’s voices requires creating environments in which children are heard within families, schools, communities, health care, and public policy. This responsibility aligns with the United Nations Convention on the Rights of the Child [45] while recognizing Indigenous understandings of children’s roles within family and community life.

#### 3.7.8. Responsibility to Uphold Self-Determination

For Indigenous children, wellness cannot be separated from Indigenous rights and self-determination. Decisions affecting Indigenous children are most effective when they are led by Indigenous Nations, communities, and organizations. Jurisdiction, governance, Indigenous-led health systems, education, and child and family services, therefore, become central conditions influencing childhood wellness.

#### 3.7.9. Responsibility for Collective Responsibility

Collectively, these responsibilities form the foundation of the Childhood Wellness Framework. Unlike cumulative risk scores or resilience inventories, the framework does not seek to classify children. Instead, it asks whether relationships, institutions, and systems are creating the conditions necessary for children to grow, belong, participate, and flourish. Childhood wellness thus becomes not simply an individual outcome but a reflection of how effectively societies fulfill their collective responsibilities to children.

#### 3.7.10. Responsibility to Future Generations

Indigenous ways of knowing remind us that responsibilities extend beyond the present generation. Decisions made today shape the lives of children yet to be born. Table 3 demonstrates the collective responsibilities, accountability questions, illustrative indicators and sources of evidence. Whereby, measuring childhood wellness requires considering sustainability, environmental stewardship, cultural continuity, and governance not only as present-day concerns but also as commitments to future generations.

Unlike ACEs and PCEs, which primarily measure experiences occurring within children’s lives, the Childhood Wellness Framework proposes measuring the fulfillment of collective responsibilities that create the conditions in which children live. Children’s wellbeing, therefore, becomes an indicator of societal accountability rather than solely an indicator of individual risk or resilience.

## 4. Discussion

The Childhood Wellness Framework is presented as an operational model for Relational Epidemiology, but its implications extend well beyond Indigenous child health. By shifting the unit of analysis from children’s experiences to the relationships, responsibilities, and systems that create the conditions of childhood, Relational Epidemiology offers a broader conceptual contribution to epidemiology, child health, public health, health systems, and policy. Rather than replacing existing approaches to measurement, it expands the purpose of epidemiological inquiry by asking not only what children experience, but also whether societies are fulfilling their collective responsibilities to children.

### 4.1. Implications for Epidemiology: Expanding What We Measure

Epidemiology has traditionally focused on describing the distribution of disease, identifying determinants of health, estimating causal relationships, and informing prevention [33,34]. Over time, the discipline has increasingly incorporated social determinants of health, recognizing that housing, education, income, racism, and environmental conditions influence health across the life course [46]. Relational Epidemiology builds upon these advances by proposing that epidemiology should also evaluate the fulfillment of collective responsibilities that shape these determinants.

This shift expands epidemiological measurement beyond documenting individual exposures and outcomes to assessing whether systems create the conditions necessary for health. Alongside measuring adverse childhood experiences or developmental outcomes, epidemiologists might evaluate access to culturally safe maternity care, family preservation, Indigenous language revitalization, clean water, housing adequacy, community infrastructure, and Indigenous-led governance. These indicators do not replace traditional epidemiological measures; rather, they provide a means of assessing whether institutions are creating environments that enable children to thrive.

Relational Epidemiology does not prescribe a single epidemiological method or single form of data. Rather, it changes the questions to which epidemiological methods may be applied. Depending upon the accountability question, future research could use longitudinal cohort designs, multilevel analyses, linked administrative and population datasets, natural experiments, policy evaluations, interrupted time-series designs, or other approaches capable of examining changes in institutional and structural conditions over time. Indigenous-led qualitative and mixed-methods approaches are equally important where relational, cultural, governance, or experiential dimensions of accountability cannot be adequately represented by standardized quantitative indicators.

Longitudinal approaches may be especially useful because both childhood development and the fulfillment of institutional responsibilities unfold over time. Such research could examine whether changes in housing policy, access to culturally safe health services, Indigenous language investment, family-preservation policy, community infrastructure, or Indigenous jurisdiction are followed by changes in the conditions experienced by children, families, and communities. Qualitative longitudinal approaches could additionally examine how relationships, belonging, cultural continuity, trust, governance, and perceptions of institutional responsibility change across time. These approaches would enable childhood wellness to be examined not only as an individual developmental outcome, but also as an indicator of changing relational and societal conditions.

### 4.2. Data Sources and Forms of Evidence

In addition, Relational Epidemiology does not prescribe a single form of data. The appropriate source of evidence follows from the responsibility and accountability question being examined. Quantitative applications may draw on population health and administrative datasets; health, education, housing, environmental, and child and family service records; policy and program data; service-access measures; fiscal and resource-allocation information; longitudinal datasets; and community- or Nation-defined indicators. Different accountability questions may therefore require different combinations of individual-, family-, community-, institutional-, policy-, and system-level information.

For example, examining the responsibility to ensure safe and healthy living conditions may involve housing quality, food security, income, service access, or infrastructure data alongside information concerning relevant policies, investments, and institutional actions. Examining responsibilities related to language, culture, land, kinship, self-determination, or belonging may require evidence that is not adequately represented in existing administrative datasets. Qualitative interviews, narratives, participatory approaches, land-based inquiry, community- and Nation-defined evidence, governance records, and mixed-methods research may therefore be equally important.

The framework does not assume that all relational knowledge should be converted into standardized variables. Some dimensions of accountability may be appropriately quantified, while others require forms of evidence capable of preserving context, relationships, meaning, and Indigenous knowledge. Methodological rigor therefore depends on selecting forms of evidence appropriate to the accountability question and on being transparent about what each source can and cannot represent.

### 4.3. Implications for Child Health Research: Asking Different Questions

The Childhood Wellness Framework also changes the questions researchers ask. Much of child health research seeks to identify risk factors, protective factors, and developmental outcomes associated with childhood experiences. These questions remain important. However, Relational Epidemiology encourages researchers to move beyond documenting children’s experiences toward examining the systems that shape those experiences.

Rather than asking why some children experience adversity, researchers might ask why some communities lack access to safe housing, culturally relevant education, Indigenous languages, or family-centered health services. Instead of focusing exclusively on resilience as an individual characteristic, research can examine how communities, institutions, and governments create environments that foster belonging, cultural continuity, and healthy development. In doing so, child health research shifts from explaining children’s outcomes to understanding the conditions through which those outcomes emerge.

#### Worked Example: Childhood Food Insecurity

Childhood food insecurity illustrates how Relational Epidemiology could extend a conventional epidemiological question without replacing existing approaches. Epidemiological studies have appropriately examined the prevalence and distribution of food insecurity, identified populations at increased risk, and evaluated associations between food insecurity and child health and developmental outcomes. These questions remain important. Relational Epidemiology adds a complementary level of inquiry by asking how the conditions producing food insecurity are created and whether those with the authority and resources to influence those conditions are fulfilling their responsibilities.

A conventional epidemiological study might ask: which children experience food insecurity, which factors are associated with increased risk, and what health outcomes are associated with that exposure? A Relational Epidemiology study would retain these questions but add: what policies, systems, resources, and institutional decisions contribute to the conditions in which childhood food insecurity occurs, who holds responsibility for changing those conditions, and are those responsibilities being fulfilled?

Operationalizing this approach would require identifying both the childhood condition and the relevant domains of responsibility. Measures of household food insecurity could therefore be examined alongside indicators of income security, food prices and accessibility, housing costs, availability of community food infrastructure, access to transportation, social-program eligibility and adequacy, and relevant policy or funding changes. In Indigenous contexts, additional questions may concern access to traditional foods, land and water, environmental conditions, the effects of jurisdictional arrangements, and the extent to which food-related programs and policies are Indigenous-led and responsive to Nation- and community-defined priorities.

Different study designs could be used depending on the accountability question. Longitudinal studies could examine whether changes in income-support policies, housing costs, food accessibility, or community infrastructure are accompanied by changes in household food insecurity over time. Natural experiments or policy evaluations could examine the effects of specific policy changes. Linked population and administrative data could identify relationships among household conditions, service access, policy environments, and child outcomes. Qualitative and mixed-methods approaches could examine how families and communities experience these systems, including barriers or forms of support that may not be visible in administrative data.

The unit of inquiry would therefore extend beyond the child or household. Relevant responsibility-holders could include governments responsible for income and housing policy, institutions responsible for service delivery, municipalities or regional systems responsible for infrastructure and transportation, and other actors with authority over food systems or environmental conditions. Importantly, collective responsibility would not imply equal responsibility. Analysis would consider differences in authority, jurisdiction, resources, obligations, and decision-making capacity when determining which actors could reasonably influence the conditions being examined.

This example also demonstrates why Relational Epidemiology is not intended to produce a single standardized accountability score. Different dimensions of food security require different forms of evidence, and causal responsibility may be distributed across interacting systems and jurisdictions. In Indigenous contexts, decisions regarding what constitutes meaningful evidence, how data are governed, and how findings are interpreted must occur within appropriate Indigenous governance structures. The purpose is therefore not simply to generate additional variables around the child, but to expand epidemiological inquiry toward the systems and decisions that create the conditions represented by those variables.

### 4.4. Implications for Clinical Practice: From Screening to Advocacy

The widespread adoption of ACEs has transformed clinical practice by encouraging trauma-informed care and early identification of adversity [36]. While screening can help clinicians recognize children and families requiring additional support, Relational Epidemiology suggests that clinical practice should extend beyond identifying risk toward addressing the conditions that produce it.

Clinicians become not only providers of treatment but also advocates for healthy childhood conditions. Screening for food insecurity, housing instability, discrimination, or social isolation should be accompanied by systems-level responses, intersectoral collaboration, and advocacy for policies that improve children’s lives [4]. Within Indigenous contexts, this includes supporting culturally safe care, Indigenous-led health services, family-centered approaches, and respect for Indigenous rights and self-determination. Evidence, therefore, becomes a tool for strengthening systems rather than simply identifying vulnerable children.

### 4.5. Implications for Public Health and Prevention: Shifting Upstream

Public health has increasingly emphasized prevention and the social determinants of health, recognizing that health is created long before illness occurs [47]. Relational Epidemiology extends this preventive orientation by focusing attention on the relationships and responsibilities that produce childhood conditions. 

Rather than directing interventions primarily toward children identified as high risk, prevention strategies can focus on strengthening families, improving housing, expanding access to Indigenous language and cultural programming, investing in early childhood services, protecting environmental health, and supporting community capacity. In this way, prevention shifts from mitigating the consequences of adversity toward creating the conditions that reduce adversity in the first place.

### 4.6. Implications for Evaluation and Measurement: Redefining Success

Evaluation frameworks frequently determine success through improvements in individual health outcomes, developmental indicators, or reductions in disease prevalence [48]. Relational Epidemiology broadens these criteria by proposing that successful interventions should also strengthen relationships, improve systems, and enhance accountability.

For example, the success of an early childhood program might be evaluated not only by improvements in developmental screening scores but also by increased family stability, greater participation in Indigenous language programs, improved access to culturally safe health services, stronger community governance, or enhanced opportunities for children’s participation. These measures recognize that healthy childhoods emerge through sustained investments in relationships and systems rather than through isolated interventions.

### 4.7. Implications for Indigenous Rights and Self-Determination: Operationalizing Accountability

For First Nations, Métis, and Inuit children, childhood wellness cannot be separated from Indigenous rights, jurisdiction, and self-determination. International and national frameworks, including the United Nations Declaration on the Rights of Indigenous Peoples [49], the United Nations Convention on the Rights of the Child [45], the Truth and Reconciliation Commission of Canada’s Calls to Action [50], the National Inquiry into Missing and Murdered Indigenous Women and Girls Calls for Justice [51] and Jordan’s Principle [52], all recognize the responsibilities of governments and institutions to uphold the rights and wellbeing of Indigenous children.

Relational Epidemiology provides a means of operationalizing these commitments. Rather than asking whether Indigenous children experience poorer health outcomes, researchers can examine whether governments, health systems, educational institutions, and child welfare systems are fulfilling their obligations to support Indigenous languages, families, cultures, lands, governance, and self-determination. Accountability, therefore, becomes measurable.

### 4.8. Implications for Policy and Governance: From Evidence to Accountability

Existing frameworks have demonstrated the importance of addressing the social and structural determinants of health [46,53]. Indigenous scholarship has further argued that colonialism constitutes a broader structural determinant shaping Indigenous health inequities [40,41,54,55]. Relational Epidemiology extends these perspectives by proposing that epidemiology evaluate not only structural determinants but also the accountability of institutions responsible for transforming them. Perhaps the most significant implication of Relational Epidemiology is its contribution to policy and governance. Epidemiological evidence has traditionally been used to describe inequities and justify policy responses. Relational Epidemiology suggests that evidence should also evaluate whether policy itself is changing the conditions responsible for those inequities.

Under this orientation, governments are no longer accountable only for reducing disparities in health outcomes; they are accountable for creating the conditions in which children can flourish. Policies affecting housing, education, income security, environmental protection, healthcare, child welfare, and Indigenous governance become measurable determinants of childhood wellness rather than background contextual factors. This shift transforms the purpose of evidence. Rather than documenting disparities after they occur, epidemiological evidence becomes a mechanism for monitoring whether societies are fulfilling their obligations to children. Accountability is therefore understood not as a political aspiration but as a measurable outcome of public policy and institutional performance.

Collectively, these implications suggest that Relational Epidemiology represents more than a new conceptual framework for child health. It offers an expanded vision of epidemiology itself one in which measurement is directed not only toward describing health outcomes but also toward evaluating whether relationships, institutions, systems, and governments are creating the conditions necessary for children, families, and communities to flourish.

### 4.9. Limitations and Ethical Considerations

Relational Epidemiology and the Childhood Wellness Framework are conceptual contributions and have not yet been empirically evaluated as a comprehensive approach to child health research. The framework is intended to reorient epidemiological inquiry toward relationships, systems, responsibilities, and accountability rather than to function as a standardized measurement instrument. Future empirical applications will therefore be required to determine how particular domains of responsibility can be examined rigorously within specific contexts, using methods appropriate to the research question, knowledge system, population, and form of evidence being considered.

A first limitation concerns the translation of relational concepts into measurable indicators. Relationships such as belonging, kinship, cultural continuity, connection to land, responsibility, and self-determination are complex and may be diminished when reduced to discrete variables. What is most easily quantified may not be what is most meaningful. Relational Epidemiology therefore does not assume that all dimensions of childhood wellness should be converted into standardized quantitative measures. Qualitative, mixed-methods, Indigenous methodological, narrative, community-defined, governance, policy, and land-based approaches may be necessary to understand dimensions of relational accountability that cannot be adequately represented through conventional epidemiological indicators.

A related concern is the risk of treating Indigenous relationality as a singular or universal framework. First Nations, Métis, and Inuit Peoples, and individual Nations and communities within these distinctions, hold diverse languages, histories, legal orders, knowledge systems, territories, governance structures, and understandings of relationship. The Childhood Wellness Framework should therefore not be interpreted as proposing a universal Indigenous model of childhood or a standardized set of indicators applicable across all Indigenous contexts. Rather, its domains identify broad relational questions whose meanings, priorities, measures, and interpretations must be determined within the relevant cultural, political, territorial, and governance context. This distinction is essential to avoid reproducing pan-Indigenous approaches that obscure the specificity of Indigenous knowledge systems.

The framework also raises questions concerning the attribution of responsibility. Childhood conditions are produced through relationships among families, communities, Nations, institutions, governments, and broader social and environmental systems, but collective responsibility does not imply equal responsibility. These actors possess different levels of authority, jurisdiction, resources, obligations, and capacity to shape childhood conditions. Families and kinship networks, for example, hold important responsibilities for care, love, and belonging, but do not possess the same authority over housing policy, income security, health-system accessibility, environmental regulation, education, or Indigenous jurisdiction as governments and institutions. Future applications of Relational Epidemiology must therefore distinguish shared responsibility from equivalent responsibility and explicitly examine who possesses the authority, resources, decision-making power, and obligations relevant to the condition under investigation.

This distinction is also necessary because accountability-oriented measures could be misapplied. Indicators intended to evaluate systems and institutions could potentially be redirected toward surveillance, classification, or deficit-based assessments of Indigenous children, families, communities, or Nations. Such use would invert the purpose of the Childhood Wellness Framework and risk reproducing the very forms of surveillance and individualization that Relational Epidemiology seeks to challenge. The framework is therefore not intended for child welfare risk scoring, family-functioning assessments, community rankings, eligibility determinations, or other applications that relocate responsibility for structurally produced conditions onto children or families. The direction of accountability must remain explicit, particularly where governments and institutions hold greater authority and resources to influence the conditions being measured.

Indigenous data sovereignty and data governance are consequently fundamental ethical considerations in any application involving First Nations, Métis, or Inuit Peoples. The identification of a potentially measurable condition does not itself create an entitlement for researchers, governments, or institutions to collect, access, link, interpret, or disseminate Indigenous data. Questions of measurement must therefore be accompanied by questions of authority: Who determines what should be measured? Who governs the data? Who interprets the findings? How may the information be used? Who benefits from its production? Data governance must also be distinctions-based and responsive to the rights, governance structures, protocols, and priorities of the Indigenous Peoples and Nations involved rather than relying on a single pan-Indigenous approach.

A particular ethical risk is that measures intended to evaluate institutional and societal responsibility could be redirected toward surveillance or deficit-based assessment of Indigenous children, families, communities, or Nations. This risk is not hypothetical. Indigenous Peoples have long been subjected to colonial systems of classification, surveillance, intervention, and child removal, often within conditions produced by structural inequities, racism, under-resourcing, and restrictions on Indigenous jurisdiction. The Childhood Wellness Framework is therefore not intended for child-welfare risk scoring, eligibility determinations, family-functioning assessments, community rankings, or other applications that relocate responsibility for structurally produced conditions onto those experiencing them. The direction of accountability must remain explicit. Where children experience inadequate housing, food insecurity, disrupted family relationships, inaccessible health services, environmental hazards, or loss of language and cultural continuity, Relational Epidemiology asks how authority, resources, jurisdiction, policy decisions, and institutional practices contributed to those conditions and whether those holding the power and obligation to act fulfilled their responsibilities. Indigenous governance over the definition, interpretation, ownership, use, and dissemination of relevant data and indicators is therefore an ethical requirement of Indigenous applications of the framework, not simply a procedural consideration.

There are also practical limitations. Existing administrative and population datasets were generally not designed to evaluate relational accountability and may inadequately capture cultural continuity, kinship, land relationships, governance, community-defined wellbeing, institutional responsibility, or self-determination. Data concerning government and institutional actions may be fragmented across jurisdictions or unavailable, while differences in definitions and data systems can complicate longitudinal or comparative analyses. These limitations highlight the importance of developing indicators with, rather than merely about, the communities and Nations whose wellbeing is being examined and of combining multiple forms of evidence where appropriate.

The development of empirically testable indicators will require further methodological work, but such development should not presume that psychometric standardization is either possible or desirable for every relational domain; validity must be considered in relation to the purpose of the measure, the form of evidence, the knowledge system, and the governance context in which it is used.

Finally, bringing Indigenous relational thought into dialog with feminist ethics of care creates both conceptual possibilities and tensions. These traditions share critiques of atomistic individualism and emphasize interdependence, relationships, care, and responsibility, but they arise from distinct intellectual, historical, political, legal, and epistemological contexts. Conceptual convergence should therefore not be interpreted as equivalence, nor should feminist ethics of care become a framework through which Indigenous relational knowledge, Indigenous rights, or self-determination must be validated. Relational Epidemiology maintains these distinctions while highlighting how both traditions challenge approaches to health that isolate individuals from the relationships and systems through which well-being is produced.

These limitations do not negate the potential contribution of Relational Epidemiology. Rather, they establish important boundaries for its development. The framework should remain responsive to context, distinctions-based in Indigenous applications, transparent about the limits of measurement, attentive to differential power and responsibility, appropriately governed, and resistant to uses that redirect accountability toward those experiencing inequitable conditions rather than toward the systems that contribute to their production.

## 5. Conclusions

For more than three decades, child health research has transformed our understanding of childhood by demonstrating that early experiences shape health across the life course. The development of Adverse Childhood Experiences (ACEs) and Positive Childhood Experiences (PCEs) fundamentally changed medicine, public health, psychology, education, and social policy. Together, these frameworks challenged the misconception that childhood simply precedes adult health; instead, they demonstrated that childhood creates it. Their contributions have been profound and enduring.

Yet every scientific framework carries assumptions about where we direct our attention. ACEs ask, “What happened to this child?” PCEs ask, “What positive experiences did this child have?” Both questions have advanced the science of child health. Both have improved our ability to recognize trauma, understand resilience, and intervene earlier in children’s lives. But both continue to place the child at the center of inquiry.

This paper has asked a different question. What if the next evolution of child health research is not to measure children more precisely, but to measure our responsibilities to them more honestly?

Relational Epidemiology does not argue that we should stop listening to children or documenting their experiences. On the contrary, children’s experiences remain among the most powerful indicators of whether societies are succeeding or failing. What changes is where we direct the scientific gaze. Instead of asking children to carry the explanatory burden for the conditions they inherit, Relational Epidemiology asks researchers to examine the relationships, institutions, systems, policies, and decisions that create those conditions. Children’s experiences become evidence, not of who they are, but of what societies have created around them.

The Childhood Wellness Framework is therefore not proposed as another screening instrument, checklist, or cumulative score. It is not another way of classifying children. It is a framework for evaluating whether families, communities, institutions, governments, and societies are fulfilling their collective responsibilities to ensure that every child is welcomed into life with dignity, grows within loving relationships, remains connected to family, language, culture, and land, and enters adulthood with hope for future generations.

For First Nations, Métis, and Inuit children, this shift is especially significant. Colonialism has repeatedly measured Indigenous children while too rarely measuring the systems that displaced their families, suppressed their languages, separated them from their lands, or denied their Nations the authority to care for them. Indigenous ways of knowing invite us to reverse this gaze, not by abandoning science, but by asking science to become accountable to the relationships and responsibilities that sustain life. In doing so, Indigenous knowledge contributes not only to Indigenous child health but to the future of epidemiology itself.

Ultimately, this paper argues that the purpose of evidence is not merely to describe inequities; it is to help transform the conditions that produce them. The success of epidemiology should not be judged solely by how accurately it predicts disease or documents adversity, but by whether it contributes to healthier relationships, stronger communities, more just institutions, and policies that enable children to flourish.

Every child enters the world through relationships. No child creates the conditions in which they are born. Those conditions are created and can be changed by the decisions adults make, the systems we build, the policies we enact, and the responsibilities we choose to honour or neglect. If childhood wellness is shaped by collective action, then our science must be capable of measuring collective responsibility.

Perhaps the most important question facing child health research is no longer, “What happened to this child?” It is, “What did we create for this child, and what will we choose to create for the next generation?” The future of child health will not be determined by increasingly sophisticated measures of adversity. It will be determined by whether we have the courage to turn the scientific gaze toward ourselves, our decisions, the institutions, policies and programs we create and the ability to take responsibility for the futures we are creating.

## Figures and Tables

**Table 1:** ACEs, PCEs, and the Childhood Wellness Framework.

	ACEs	PCEs	Childhood Wellness Framework
Primary Question	What adverse experiences did this child experience?	What positive experiences did this child experience?	What conditions and collective responsibilities enable children to flourish?
Primary Unit of Analysis	Child	Child	Relationships, systems, and collective responsibilities
Focus of Measurement	Adversity	Positive experiences and resilience	Conditions for childhood wellness and societal accountability
Theoretical Orientation	Life course epidemiology; trauma	Resilience and protective factors	Relational Epidemiology
Purpose	Identify risk	Identify strengths	Evaluate whether societies are fulfilling their responsibilities to children
Primary Outcome	Risk of poor health	Positive health and resilience	Childhood wellness as an indicator of accountable systems
Primary Users	Clinicians, researchers, public health	Clinicians, researchers, public health	Researchers, clinicians, governments, Indigenous Nations, policymakers, health systems

**Table 2:** The Childhood Wellness Framework as an operational model for Relational Epidemiology.

Framework Component	Description	Implications for Research and Practice
Theoretical Foundation	Relational Epidemiology conceptualizes childhood as emerging through relationships, systems, and collective responsibilities rather than solely through individual experiences.	Shifts the unit of analysis from children to the relational, social, cultural, environmental, and political conditions that shape childhood.
Epistemological Foundations	Grounded in Indigenous ways of knowing and feminist ethics of care, which view children as inherently relational and embedded within families, communities, cultures, lands, and future generations.	Encourages research that values reciprocity, relational accountability, collective wellbeing, and self-determination.
Purpose	To evaluate whether societies are creating the conditions necessary for children to thrive rather than only documenting adversity or resilience.	Supports accountability-oriented approaches to child health research, policy, and practice.
Unit of Analysis	Relationships, responsibilities, systems, institutions, and environments that shape childhood.	Expands epidemiological inquiry beyond individual risk factors toward structural and relational accountability.
Conceptual Focus	Childhood wellness emerges through the fulfillment of collective responsibilities rather than individual protective factors.	Reframes child wellbeing as an indicator of societal conditions rather than solely an individual outcome.
Collective Responsibilities	Welcome life; nurture love and belonging; strengthen families and kinship; sustain language, identity, and culture; protect relationships with land and environment; ensure safety and basic needs; honor children’s voices; uphold self-determination; support community wellbeing; protect future generations.	Provides a conceptual framework for identifying conditions that support healthy child development and collective wellbeing.
Primary Responsibility Holders	Families, kinship networks, communities, Nations, institutions, health systems, education systems, governments, and society.	Clarifies shared accountability for creating the conditions that enable childhood wellness.
Applications	Child health research, epidemiology, clinical practice, public health, program evaluation, policy, and Indigenous self-determination.	Guides the development of accountability indicators, systems evaluation, and strengths-based approaches to childhood wellness.

**Table 3:** Childhood Wellness Framework: collective responsibilities, accountability questions, illustrative indicator families, and sources of evidence.

Collective Responsibility	Guiding Accountability Question	Illustrative Indicator Families	Potential Sources of Evidence	Primary Responsibility Holders
Responsibility to Welcome Life	Are children welcomed into life with dignity, safety, and culturally appropriate care?	Access to prenatal and maternity care; culturally safe care; Indigenous midwifery; opportunities for birth close to home; maternal wellbeing; continuity of care; birth outcomes	Health administrative data; maternity-service records; community- or Nation-defined indicators; patient and family experience data; qualitative accounts of pregnancy and birth	Families; communities and Nations; maternity care providers; health systems; governments
Responsibility to Nurture Love and Belonging	Do children grow within stable, caring relationships and experience love, belonging, and inclusion?	Caregiver continuity; family stability; opportunities for secure relationships; family preservation; community belonging; experiences of inclusion, discrimination, or racism	Longitudinal child and family surveys; community-defined measures; child and family narratives; school and community data; child welfare records where appropriate	Families and kinship networks; communities; schools; health systems; child and family services
Responsibility to Strengthen Families and Kinship	Are children supported within healthy family, kinship, and intergenerational relationships?	Kinship care; family reunification and preservation; multigenerational supports; caregiver wellbeing; housing stability; access to family support; continuity of extended kin relationships	Child and family service records; housing and social program data; community and Nation records; program evaluations; qualitative and family-defined evidence	Families and kinship networks; communities and Nations; child and family services; health and social systems; governments
Responsibility to Sustain Language, Identity, and Culture	Do children have meaningful opportunities to learn and live their languages, cultures, histories, and identities?	Availability and participation in Indigenous language programming; opportunities for cultural participation; Elders’ involvement; culturally relevant education; identity affirmation; investments in language and cultural continuity	Nation and community data; education records; language and cultural program data; policy and funding records; community-defined indicators; qualitative accounts	Families; Elders and knowledge holders; communities and Nations; education systems; cultural organizations; governments
Responsibility to Protect Relationships with Land and Environment	Do children have safe, meaningful, and sustained relationships with land, water, food systems, and the natural environment?	Safe drinking water; environmental quality; access to land and water; traditional food access; opportunities for land-based learning; environmental stewardship; exposure to environmental hazards	Environmental monitoring; water-quality data; land-use and access information; food-system data; Nation and community records; land-based and community-defined knowledge	Nations; communities; governments; environmental agencies; industry where applicable; land and resource management systems
Responsibility to Ensure Safety and the Conditions for Daily Living	Are the material, social, and service conditions necessary for healthy childhood consistently available and accessible?	Housing quality and stability; food security; income security; access to health care and education; transportation; recreation; digital connectivity; community safety	Population and administrative data; housing and income data; health- and education-service records; service-access mapping; policy and program data; community-defined evidence	Governments; municipalities; communities and Nations; health systems; education systems; housing and social-service systems
Responsibility to Honor Children’s Voices	Are children able to participate meaningfully in decisions affecting their lives in ways appropriate to their age, development, culture, and relationships?	Opportunities for child and youth participation; youth advisory structures; participation in health, education, community, and policy decisions; experiences of being heard and respected	Youth advisory records; school and health-system experience data; policy and program documentation; participatory research; interviews, talking circles, and other culturally appropriate approaches	Families; schools; health systems; communities and Nations; child and family services; governments
Responsibility to Uphold Self-Determination	Are decisions affecting Indigenous children governed and led by Indigenous peoples, Nations, and communities?	Indigenous jurisdiction and governance; Indigenous-led health, education, and child and family services; authority over funding and program design; implementation of Indigenous rights; Nation-defined priorities and accountability mechanisms	Legislation; governance agreements; policy documents; fiscal and funding data; administrative records; Nation and community governance records; Indigenous-led evaluation	Indigenous Nations and governments; federal, provincial, territorial, and other governments; institutions and service systems
Responsibility to Support Community and Collective Wellbeing	Are communities and Nations supported to create the social, cultural, economic, and infrastructural conditions in which children can flourish?	Community infrastructure; cultural and recreational programming; service capacity; community safety; social connectedness; local leadership and governance capacity; investment in community-defined priorities	Community and Nation plans; infrastructure and service data; fiscal and investment records; community-defined wellbeing indicators; program evaluations; qualitative and mixed-methods evidence	Communities and Nations; governments; municipalities; institutions; civil society and service organizations
Responsibility to Future Generations	Do present-day decisions protect and strengthen the conditions required for future generations to flourish?	Long-term environmental stewardship; language and cultural continuity; sustainability; intergenerational wellbeing; long-term policy investment; protection of land and resources; continuity of Indigenous governance and knowledge	Longitudinal environmental and population data; policy and budget commitments; land and resource planning; language and cultural continuity data; Nation-defined intergenerational indicators; qualitative and land-based knowledge	Families and kinship networks; communities and Nations; governments; institutions; industry where applicable; society

Note: The examples presented in this table are illustrative rather than standardized or validated measures. Each collective responsibility represents a broad domain of accountability that requires further contextual definition before empirical investigation. Selection of indicators and sources of evidence should follow the specific accountability question, responsibility-holder, research context, and methodological approach. Quantitative indicators may be appropriate for some questions, while qualitative, mixed-methods, policy, governance, community-defined, Indigenous methodological, or land-based forms of evidence may be necessary for others. In Indigenous contexts, definitions, indicators, benchmarks, data sources, interpretation, and use must be distinctions-based and governed according to the rights, priorities, knowledge systems, and data-governance requirements of the relevant First Nations, Métis, Inuit, Nations, or communities. The framework is not intended to produce a universal childhood wellness score or to rank children, families, communities, or Nations.

## Data Availability

The original contributions presented in this study are included in the article. Further inquiries can be directed to the corresponding author.

## Abbreviations

The following abbreviations are used in this manuscript:

CWF	Child Wellness Framework
